# Anti-West Nile virus activity of *in vitro *expanded human primary natural killer cells

**DOI:** 10.1186/1471-2172-11-3

**Published:** 2010-01-20

**Authors:** Mingjie Zhang, Sylvester Daniel, Yong Huang, Caren Chancey, Qingsheng Huang, Ying F Lei, Andriyan Grinev, Howard Mostowski, Maria Rios, Andrew Dayton

**Affiliations:** 1 Laboratory of Molecular Virology, Center for Biologics Evaluation and Research, Food and Drug Administration, 1401 Rockville Pike, Rockville, MD 20892, USA; 2 Cellular and Tissue Therapy Branch, Center for Biologics Evaluation and Research, Food and Drug Administration, 1401 Rockville Pike, Rockville, MD 20892, USA; 3 Department of Life Science, Northwestern Polytechnical University, 127 Youyi Xilu, Xi'an, Shaanxi 710072, China; 4 Department of Microbiology, The Fourth Military Medical University, Xi'an, Shaanxi 710032, China

## Abstract

**Background:**

Natural Killer (NK) cells are a crucial component of the host innate immune system with anti-viral and anti-cancer properties. However, the role of NK cells in West Nile virus (WNV) infection is controversial, with reported effects ranging from active suppression of virus to no effect at all. It was previously shown that K562-mb15-41BBL (K562D2) cells, which express IL-15 and 4-1BBL on the K562 cell surface, were able to expand and activate human primary NK cells of normal peripheral blood mononuclear cells (PBMC). The expanded NK cells were tested for their ability to inhibit WNV infection *in vitro*.

**Results:**

Co-culture of PBMC with irradiated K562D2 cells expanded the NK cell number by 2-3 logs in 2-3 weeks, with more than 90% purity; upregulated NK cell surface activation receptors; downregulated inhibitory receptors; and boosted interferon gamma (IFN-γ) production by ~33 fold. The expanded NK (D2NK) cell has strong natural killing activity against both K562 and Vero cells, and killed the WNV infected Vero cells through antibody-dependent cellular cytotoxicity (ADCC). The D2NK cell culture supernatants inhibited both WNV replication and WNV induced cytopathic effect (CPE) in Vero cells when added before or after infection. Anti-IFN-γ neutralizing antibody blocked the NK supernatant-mediated anti-WNV effect, demonstrating a noncytolytic activity mediated through IFN-γ.

**Conclusions:**

Co-culture of PBMC with K562D2 stimulatory cells is an efficient technique to prepare large quantities of pure and active NK cells, and these expanded NK cells inhibited WNV infection of Vero cells through both cytolytic and noncytolytic activities, which may imply a potential role of NK cells in combating WNV infection.

## Background

Natural killer (NK) cells are able to kill viral infected cells directly and produce inflammatory cytokines that limit infection. NK cell activation is controlled by the integration of signals from activation and inhibitory receptors. The NK cells from normal blood donors are usually in inhibitory states, but can be activated, either directly or indirectly, through CD4^+ ^T cells, dendritic cells (DC), monocytes/macrophages, or NKT cells. Interferons, and macrophage-derived cytokines, including IL-1β, IL-2, IL-12, IL-15, IL-18, and TNF-α can contribute to NK cell activation directly in a MHC class I independent manner [1].

NK cells should have anti-WNV properties. However, surprisingly few experiments have been published describing the antiviral activity of NK cells against WNV or other flaviviruses [2]. Knowledge about NK cells in WNV infection is limited to the analysis of NK cell activity during WNV infections in humans and NK cell depleted mice. Infection of mice with WNV transiently activates and then suppresses NK cell activity [3]. WNV infection may attenuate NK cell cytotoxicity by increasing cell surface expression of MHC class I molecules [4-6] to overcome susceptibility to NK cell mediated lysis. Splenocytes from WNV immunized mice have poor NK cell lytic activity [7]. Mice genetically deficient in NK cells or with NK cells depleted by anti-NK cell antibody demonstrate no increased morbidity or mortality for WNV infection when compared to wild type controls [2,8]. Thus, at least for WNV infection in mice, NK cells appear to be dispensable for controlling infection and disease, despite their well documented role in combating viral infection in general.

Presumably NK cell knockout or NK cell depletion does not promote WNV infection of mice because NK cell functions are effectively inhibited by WNV. NK cells may be able to control WNV infection if this inhibition is alleviated or bypassed. Encouraged by recent advancements in cancer treatment with NK cells [9-11], expanded, activated NK cells from human peripheral blood mononuclear cells (PBMC) *in vitro *were prepared, and tested for the ability to inhibit WNV in tissue culture. The *in vitro *expanded NK cells were demonstrated to inhibit WNV infection of Vero cells efficiently. This underscores the potential importance of NK cells in controlling WNV infection.

## Results

### Co-culture with radiation killed stimulating cells in vitro significantly expanded NK cells in human PBMC

**Figure 1:**
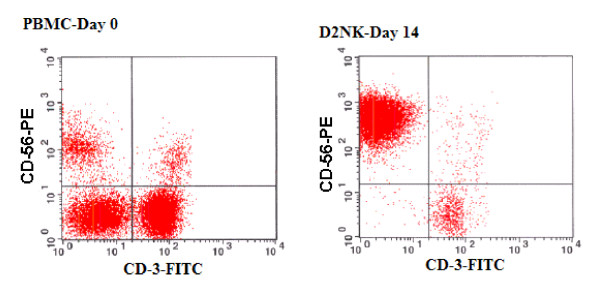
**In vitro expansion of PBMC enriched CD56+ cells**. NK cells before and 2 weeks after expansion by co-culture with K562-mb15-41BBL (D2NK) cells were stained with anti-CD56-PE and anti-CD3-FITC antibodies as the markers for NK cells and T cells, and then analyzed by flow cytometry. The UL quadrant counts (NK cells) are 9.60% and 91.20% for PBMC-Day 0, D2NK-Day 14 respectively; The LR quadrant counts (T cells) are 53.22% and 6.60% for PBMC-Day 0, D2NK-Day 14 respectively. PBMC isolated from the Buffy coat were either cultured freshly, or after frozen storage in liquid nitrogen, with equivalent results. The data presented in this figure is typical of 12 expansions made from the PBMC of 5 different donors. Averaged over these 12 experiments, NK cells were 10.05% ± 4.42 and T cells 52.44% ± 6.71 CD3+ before expansion. After expansion, NK cells were 85.25% ± 12.53 and T cells 12.12% ± 5.52 CD3+.

In co-cultures with 1 × 10^7 ^lethally radiated K562-mb15-41BBL (K562D2) stimulating cells *in vitro*, 1 × 10^7 ^PBMC were expanded to 1 × 10^8 ^in 2 weeks. CD56^+ ^(a NK cell marker) and CD3^+ ^(a T cell marker) cells changed from 9.60% and 53.22% before expansion to 91.20% and 6.60% respectively after expansion (Figure 1). The absolute CD56^+ ^cell number increased from about 1 million to 100 million, or about 100 fold. The absolute CD3^+ ^cell number remained the same, but the CD3^+^/CD56^+ ^ratio changed from about 5.5 before expansion to about 0.07 after expansion, or a decrease of about 2 logs. Since culture medium alone has been well documented to give only about a 2- to 5-fold expansion of CD56^+^CD3^- ^NK cells [12], a control for this was not included in this experiment. K562D2 stimulatory cells disintegrate and disappear over about 7 days in the culture. The results in this paper represent more the 40 expansions with fresh or frozen PBMC.

### Expanded NK cells are cytolytic through both natural killing and ADCC

**Figure 2:**
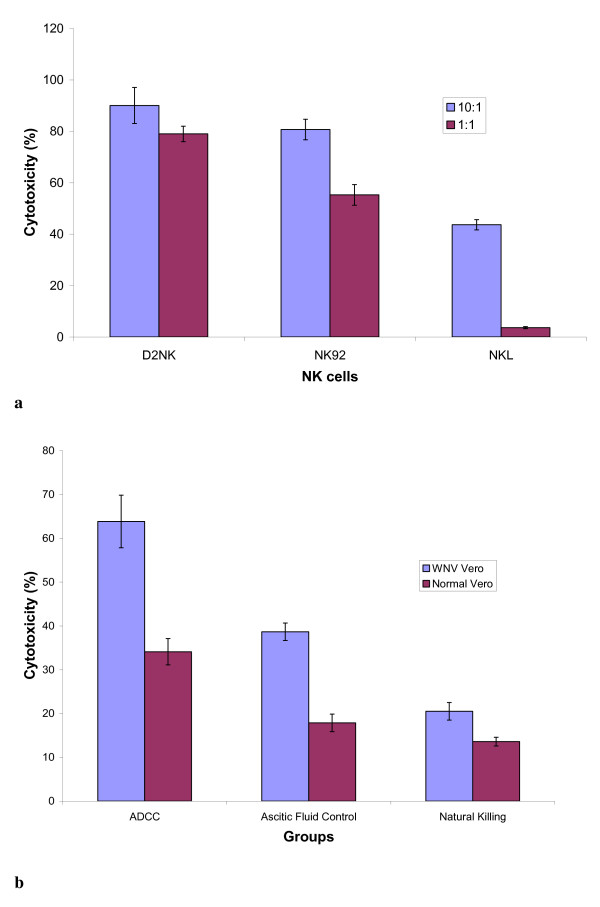
**Cytotoxicity of the D2NK cells**. (a) Natural killing activities of NK92, NKL cell lines and in vitro expanded primary D2NK cells. The effector cells were NK92, NKL, D2NK cells, the target cells were K562 cells; the effector: target ratio were 10:1, and 1:1; reaction at 37°C for 4 hours. (b) Natural killing and ADCC of D2NK cells against normal and WNV infected Vero cells. The ADCC group was done with effector cell, target cells, and WNV specific hyper-immune ascetic fluid; the natural killing group was done with effector cells, target cells, but no antibody; the Ab control group was done with effector cell, target cells, and HIV-1 RT specific ascetic fluid as nonspecific antibody and ascetic fluid control; the target cells were Vero cells either uninfected or 48 hours post infection with 100 TCID50 units of WNV; the effector cells were in vitro expanded D2NK cells; the E:T ratio was 10:1; WNV hyper-immune acetic fluid and HIV-1 RT McAb in acetic fluid were used at 1:1000 dilutions for ADCC; All reactions were performed in triplicate. The SD is the standard deviation from 6 tests with NK cells expanded from 6 different donor PBMC.

D2NK cells expanded by co-culture with K562D2 stimulatory cells have been reported to have enhanced cytolytic function against B-lineage cell lines [12]. In this study, K562 and Vero cells were used as target cells because K562 is a standard target cell for NK cell cytotoxicity assay and Vero cells were used for WNV infection and anti-WNV assays in our protocols. In a 4-hour cytotoxicity assay, D2NK cells expanded with K562D2 showed very strong cytotoxicity, compared to NK92 and NKL cell lines [12], against uninfected K562 target cells at effector:target ratios ranging from 10:1 to 1:1 (Figure 2a). As shown in Figure 2b, D2NK cells were cytotoxic to both normal Vero cells and WNV infected Vero cells, and WNV infection made the Vero cells more susceptible to D2NK killing. Antibody from WNV-hyperimmune ascetic fluid enhanced D2NK cytotoxicity to WNV infected Vero cells by greater than two-fold at the 10:1 E:T ratio tested. Control ascetic fluid containing unrelated antibody (HIV-1 RT specific McAb) had no effect on killing (Figure 2b, "Ab Control), excluding the possibility of nonspecific killing by IgG or other components of ascetic fluid in the ADCC assay. This result represents 6 cytotoxicity assays performed.

### Expanded NK cells are activated

**Figure 3:**
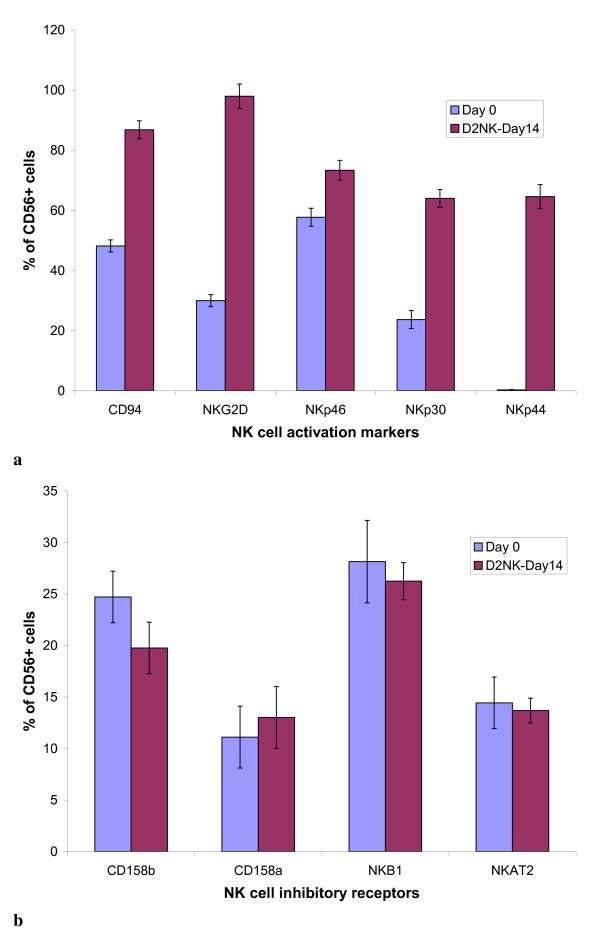
**Effects of in vitro expansion on expression of NK cell receptors**. D2NK cells were stained with PE or FITC conjugated antibodies against (a) cell activating receptors, NKG2D, NKp30, NKp44, NKp46 NK, or (b) cell inhibitory receptors, CD158a, CD158b, NKB1, NKAT2 NK, and then analyzed by flow cytometry. CD94 can be either an activation or inhibitory KIR, according to which downstream receptor it activates. The data presented represent the combined results from two donors, one tested in singlicate, another tested in duplicate. The error bars represent standard deviations, according equal weight to all three assays.

NK cell related surface receptors other than CD56 and CD3 were also assessed by FACS. NKG2D, NKp30, NKp44, and NKp46 are NK cell activation receptors; CD158a, CD158b, NKB1, and NKAT2 are NK cell inhibitory receptors [13-15]. The results from 2 donors are shown in Figure 3. The expression rates of the activation receptors (Figure 3a) on the NK cells expanded using K562D2 are much higher than on the PBMC before expansion. For example, NKG2D^+^/CD56^+ ^cells increased from 30% in PBMC to more than 95% in D2NK. However, D2NK cells had little influence on expression of inhibitory receptors (Figure 3b), with the exception of a reduction of CD158b. CD94^+^/CD56^+^, an activating or inhibitory KIR (depending upon which downstream receptor it activates) increased from 48% in PBMC to 86% in D2NK (Figure 3a).

Significant changes in the expression of cytolytic receptors during a number of co-culture experiments were observed. The inhibitory CD158b^+^/CD56^+^cells were reduced from 25% in PBMC to 20% in D2NK (Figure 3b); whereas the activating NKp30^+^/CD56^+^cells increased from 24% in PBMC to 64% in D2NK, and the activating NKp46^+^/CD56^+^cells increased from 58% in PBMC to 73% in D2NK (Figure 3a). The most significant change was the activating NKp44^+^/CD56^+ ^expression, from an almost undetectable 0.31% in PBMC to 65% in D2NK after expansion. This corresponded to a more than 208 fold increase (Figure 3a).

**Figure 4:**
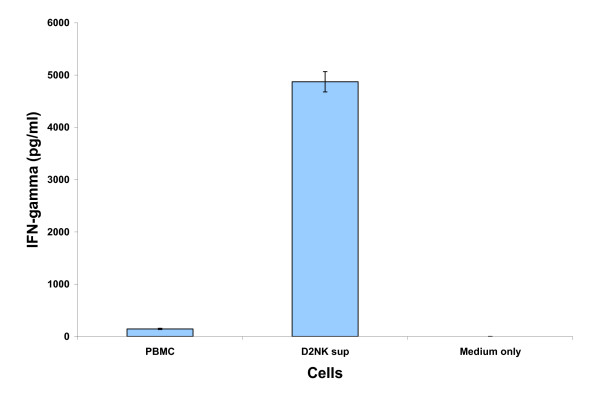
**IFN-γ production of the expanded NK cells**. Supernatants from 6 × 10^6 ^PBMC, D2NK cells cultured for 48 hours at a 6-well plate in 2 ml medium were tested with human IFN-γ EIA kit. The concentrations of IFN-γ from PBMC and D2NK are 154 ± 12 pg/ml, and 5052 ± 493 pg/ml respectively; the culture medium has no detectable human IFN-γ. The PBMC and D2NK from 3 different donors were tested in triplicate for each. The error bars represent SD.

Because upregulated IFN-γ production is another important characteristic of activated NK cells, The IFN-γ levels in PBMC and D2NK cell culture supernatants were measured. In 2 ml cultures containing 2 × 10^6 ^cells each, D2NK cells produced 4871 pg/ml vs. 154 pg/ml IFN-γ produced by PBMC in 48 hours, or about 33 fold more IFN-γ produced by D2NK cells than normal PBMC (Figure 4).

### NK cells and NK cell culture supernatants inhibited WNV replication in Vero cells

**Figure 5:**
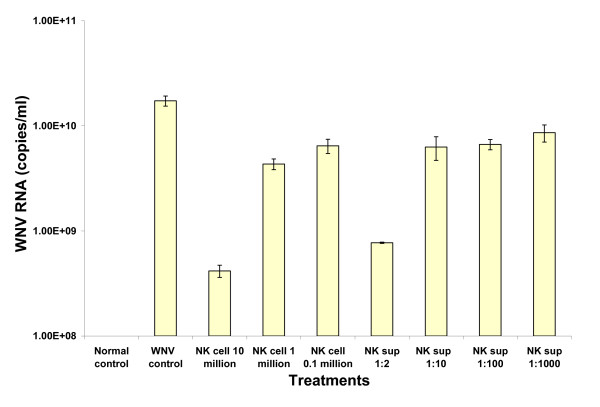
**NK cells and supernatants inhibited WNV replication in Vero cells**. NK cells or culture supernatants were added to WNV-infected or control Vero cultures 2 hours post infection. Supernatants from testing and control Vero cell cultures were collected on day 10 post WNV infection, and WNV RNA was extracted with Qiagen viral extraction kit and then analyzed with TaqMan RT-PCR. The NK cell culture supernatant was from D2NK cells.

As shown in table 1, D2NK cells at 10 million/T25 flask, and NK cell culture supernatants at a 1:2 dilution completely inhibited WNV induced CPE in Vero cells until day 10 post-infection when cultures were terminated. NK cells at 1 million/T25 flask, and NK cell culture supernatants at 1:10 dilution significantly delayed the WNV induced CPE in Vero cells. In control experiments, neither NK cells nor NK cell culture supernatants at the concentrations tested induced CPE in Vero cells (data not shown). The inhibitory effects of NK cells and NK cell culture supernatants on WNV replication in Vero cells were further confirmed with TaqMan RT-PCR of viral RNA extracted from the culture supernatants collected on day 10 post infection (Figure 5). The NK cells reduced WNV viral load in a cell number dependent manner, and supernatants reduced the WNV viral load in a dose dependent manner.

**Table 1:** D2NK cells and D2NK cell culture supernatants inhibited WNV induced CPE in Vero cells.

Days Post Treatment	1	2	3	4	5	7	8	9	10
Normal control	-	-	-	-	-	-	-	-	-
WNV control	-	-	+	+	3+	4+	4+	4+	4+
NK cells 10 million	-	-	-	-	-	-	-	-	-
NK cells 1 million	-	-	-	-	+	4+	4+	4+	4+
NK cells 0.1 million	-	-	-	+	2+	4+	4+	4+	4+
NK sup 1:2	-	-	-	-	-	-	-	-	-
NK sup 1:10	-	-	-	-	+	4+	4+	4+	4+
NK sup 1:100	-	-	-	+	2+	4+	4+	4+	4+
NK sup1:1000	-	-	-	+	3+	4+	4+	4+	4+

### Anti-IFN-γ antibody neutralized NK cell supernatant inhibition of WNV replication in Vero cells

**Figure 6:**
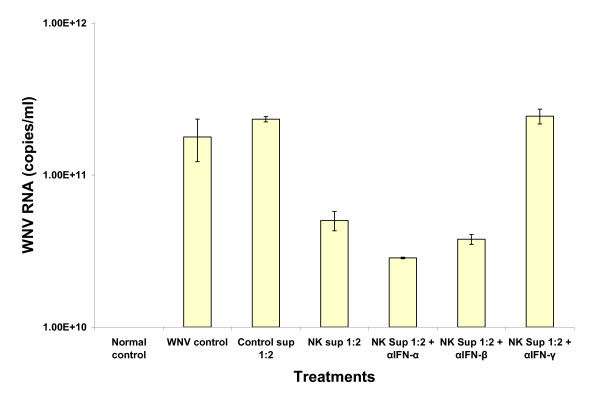
**Anti-IFN-γ antibody neutralized NK cell supernatant inhibition of WNV replication in Vero cells**. NK culture supernatants with and without antibodies to IFNs were added to WNV-infected or control Vero cultures 2 hours post infection. Supernatants from testing and control Vero cell cultures were collected 10 days post WNV infection, and WNV RNA was extracted with Qiagen viral extraction kit and then analyzed with TaqMan RT-PCR. The NK cell culture supernatant was from D2NK cells.

When anti-IFN antibodies were individually pre-incubated with D2NK cell culture supernatant, only anti-IFN-γ inhibited the ability of NK cell supernatants to suppress WNV infection of Vero cells (Table 2). In control experiments, supernatant from the irradiated K562D2 cells alone had no inhibitory effect on WNV infection of Vero cells (Table 2, Figure 6).

**Table 2:** Anti-IFN-γ antibody blocks D2NK supernatant inhibition of WNV CPE in Vero cells.

Days Post Treatments	1	2	3	5	6	7	8	9	12	13
Normal control	-	-	-	-	-	-	-	-	-	-
WNV control	-	-	-	2+	4+	4+	4+	4+	4+	4+
Control Sup 1:2	-	-	-	2+	3+	4+	4+	4+	4+	4+
NK Sup 1:2	-	-	-	-	-	-	-	-	-	-
NK Sup 1:2 + α IFN-α^a^	-	-	-	-	-	-	-	-	-	-
NK Sup 1:2 + α IFN-β^a^	-	-	-	-	-	-	-	-	-	-
NK Sup 1:2 + α IFN-γ^a^	-	-	-	1+	2+	3+	3+	4+	4+	4+

The diluted D2NK cell culture supernatant was incubated with anti-IFN-α, anti-IFN-β, or anti-IFN-γ antibody at 37°C for 1 hour before being applied to the WNV infected Vero cells.

### Pretreatment with NK cell supernatants made Vero cells resistant to WNV infection

**Figure 7:**
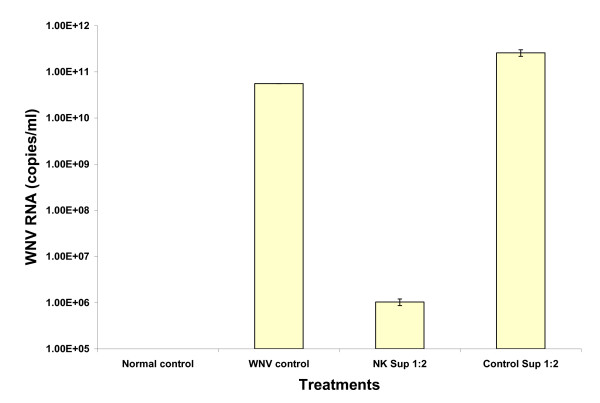
**NK cell supernatant pretreatment made Vero cells resistant to WNV infection**. Vero cell cultures were treated with NK cell culture supernatants or control supernatants overnight prior to infection with WNV. Supernatants from testing and control Vero cell cultures were collected 10 days post WNV infection, and WNV RNA was extracted with Qiagen viral extraction kit and then analyzed with TaqMan RT-PCR. The NK cell culture supernatant was from D2NK cells.

Pretreatment of Vero cells with D2NK cell supernatant for 24 hours, followed by washing and WNV infection reduced the supernatant WNV RNA copy numbers by ~4 logs as measured by TaqMan RT-PCR on day 10 post infection. By comparison, pretreatment with control supernatants had no inhibitory effect on WNV RNA copy numbers (Figure 7).

## Discussion

NK cells are activated during a wide variety of viral infections by virus-induced type I IFNs. The importance of the NK cell defense against virus infections is highlighted by the susceptibility of mice depleted of NK cells to many experimental infections and by the invasive or disseminated viral disease that is associated with naturally occurring NK cell deficiencies in humans [16]. However, many viruses, including WNV, have developed mechanisms to evade the NK cell response. These mechanisms include expression of MHC class I homologs encoded by viruses, selective modulation of MHC class I protein expression, inhibition of activating receptor function and production of cytokine-binding proteins or cytokine-receptor antagonists encoded by viruses [17]. These mechanisms also include direct viral effects on NK cells, such as infection of NK cells and viral envelope protein blockage of non-class I NK cell inhibitory receptors [2-4,6,17]. Mice genetically deficient in NK cells or depleted of NK cells by anti-NK cell antibody demonstrated no increased morbidity or mortality compared to controls when infected with WNV [2,8]. However, it would seem that this apparent lack of NK protection is only because WNV normally suppresses or bypasses the NK response. Therefore, it is reasonable to expect that blocking the viral evasion process and reinforcing NK cell function might lead to improved control and accelerated clearance of the viral infection.

Co-culture of PBMC with K562D2 not only expanded NK cells over a hundred fold, but also activated NK cells. All of the activating receptors tested including NKG2D, NKp30, NKp44, and NKp46 were upregulated after expansion. Most noticeably, NK cells expressing the natural cytotoxicity receptor, NKp44, were up regulated from only 0.31% before to 64.59% after expanding, which corresponds to a greater than 200-fold increase. Consistent with the upregulated natural killing receptors, the D2NK cells are more potent in killing K562 target cells (Figure 2a) than either the NK92 or NKL cell lines. The inhibitory markers in D2NK cells showed very small changes, although CD158b appeared to be modestly downregulated. Further studies would be required to determine whether the small CD158a downregulation is statistically significant. The upregulation of the activation receptors combined with the possible downregulation of one of the inhibitory receptors suggest an overall bias towards an activation profile in D2NK cells. The upregulation of the IFN-γ production of D2NK cells (Figure 4) with no detectable IL-10 (data not shown) both before and after expansion further confirmed the activation status of the D2NK cells. We have tried to measure TGF-β production by D2NK, but have so far been unable to detect TGF-β above the high background concentration of TGF-β in the culture medium. We have not yet attempted to expand NK cells in serum-free medium.

The NK cell lines, NK92 and NKL, have efficacy disadvantages compared to D2NK. Both cell lines have less natural killing activity than D2NK (Figure 2a). NK92 has no ADCC activity because it lacks FcR and NKL has only weak ADCC in our assays (not shown). D2NK shows strong ADCC (Figure 2b). ADCC activity has been shown to play an important role in anti-cancer therapy and in the light of data presented here may warrant assessment for function in combating WNV infection. The WNV infected Vero cells are more susceptible to D2NK killings than normal Vero cells by both ADCC and natural killing. It should be interesting to further study the underneath mechanisms.

The inhibition of WNV replication by NK cells delivered prior or post infection observed in this study, suggests the possibility of controlling WNV infection by adoptive transfer of NK cells expanded in vitro. It remains to be determined whether the direct cytotoxicity and ADCC or the endogenous production of IFN-γ afforded by these expanded NK cells provides advantages over alternative treatments for WNV such as direct administration of IFN-γ.

## Conclusions

Co-culture of human PBMC with irradiated K562D2 stimulatory cells together with medium supplemented with IL-2 efficiently expanded and activated NK cells, as measured by IFN-γ production, direct cytotoxicity, ADCC and surface expression of NK activation markers. The *in vitro *expanded D2NK cells and their culture supernatant inhibited WNV infection of Vero cells. These results help characterize the potential role of NK cells in WNV infection and demonstrate that NK cells are capable of combating WNV infections in the right conditions.

## Methods

### K562D2 Stimulatory cells

The co-stimulatory cell line, K562-mb15-41BBL (K562D2), expressing IL-15 and 4-1BB ligand on K562 cell surface [12], was kindly provided by Dr. Dario Campana.

### NK cells and NK cell culture supernatants preparation

Human PBMC were isolated from Buffy coats (from the NIH blood bank) by Histopaque (Sigma, St. Louis, MO), then incubated with equal numbers of lethally irradiated (10,000 rads) stimulating cells in the presence of 10 IU/ml human IL-2 (National Cancer Institute BRB Preclinical Repository, Rockville, MD) in RPMI 1640 and 10% FCS according to Imai C et al. [12]. The co-culture lasted for 2-3 weeks, with a 50% media change every 2 days after the first week. D2NK (NK cells expanded with K562D2 stimulatory cells) and their supernatants were collected 48 h after the last media change by centrifugation; NK cells were washed once with PBS before analysis, or application to WNV infected Vero cells for anti-WNV assays.

The PBMC before expansion and the expanded D2NK cells were all independently analyzed by FACS for surface receptors after staining with antibody conjugates including hCD3-FITC, hCD16-FITC, hCD56-PE, hCD158a-PE, hCD185b-FITC, hCD94-FITC, hCD158e1 (NKB1)-FITC, hCD158b1/b2 (KIR-NKAT2)-PE (BD Biosciences, San Diego, CA); and NKG2D-PE, NKp30-PE, NKp46-PE, NKp44-PE (Coulter, Fullerton, CA).

Supernatants from co-cultivated NK and from the control (stimulatory cells alone, without PBMC) were independently filtered through 0.2 um filters, and either used immediately or stored at -70°C in aliquots. In order to measure the IFN-γ concentration with EIA kit (R&D Systems, Minneapolis, MN) in the supernatants, 6 × 10^6 ^PBMC, D2NK cells were replated into a single well of 6-well plate in 2 ml of the NK cells culture medium, and cultured for 48 h at 37°C before harvest.

### Cytotoxicity assays

The CytoTox 96^® ^Non-Radioactive Cytotoxicity Assay kit from Promega (Madison, WI) was used to measure the direct cytotoxicity of the NK cells. K562 cells, normal Vero cells and Vero cells 48 hours after 100 TCID50 of WNV strain FDA/HU-02 (GB# AY646354) infection were used as the target cells. Target cells at 2.5 × 10^4 ^were dispensed into 96-well U-bottom plates (Becton Dickinson, San Jose, CA) in triplicate. Effector: target cells ratios of 10:1 and 1:1 were mixed in a final volume of 200 μl per well and incubated for 4 hours at 37°C in a 5% CO2 incubator. Anti-WNV antibody of WNV hyperimmune ascetic fluid from ATCC (Rockville, MD) was used at 1:1000 dilutions for ADCC assay, anti-HIV-1 RT McAb in ascetic fluid (catalog # 2483) from NIH AIDS Research & Reference Reagent Program (Gaithersburg, MD) was used as the negative control. The following formula was used to calculate the percent cytotoxicity: % Cytotoxicity = (Experimental - Effector Spontaneous - Target Spontaneous) 100/(Target Maximum - Target Spontaneous).

### WNV infection of Vero cells

1 × 10^6 ^Vero cells were cultured in T25 flasks, infected with 100 TCID50 of WNV strain FDA/HU-02 (GB# AY646354)[16] at 37°C for 2 h, then cultured at 37°C in a 5% CO_2 _incubator, and observed for CPE daily. Culture supernatants were collected at indicated time points for WNV viral RNA extraction and TaqMan RT-PCR [17].

### NK cell and NK cell culture supernatant treatment of WNV infected Vero cells

D2NK cells at different concentrations and D2NK cell culture supernatants at different dilutions were added to the WNV infected Vero cells in T25 flasks in duplicate and observed daily for CPE. Culture supernatants were collected for WNV viral RNA extraction and TaqMan RT-PCR [17] analysis at day 7. The control supernatants were collected from lethally irradiated stimulatory cell alone cultures without PBMC.

### NK cell culture supernatant treatment with anti-IFN antibodies

IFN-α, IFN-β, IFN-γ (NIAID Reference Reagent Repository, Gaithersburg, MD) were tested in WNV infection of Vero cells, and confirmed all 3 IFNs were able to inhibit WNV replication; anti-IFN-α antibody, anti-IFN-β antibody, and anti-IFN-γ antibody (NIAID Reference Reagent Repository, Gaithersburg, MD) were tested and confirmed of their neutralization activities against corresponding IFN. D2NK cell culture supernatants were diluted 1:2 with culture medium, and supplemented with anti-IFN antibodies individually at a dilution of 1:1000, incubated at 37°C for 1 h before addition to Vero cells infected with WNV for 2 h in duplicate to test which antibody could neutralize the anti-WNV activity. Infected Vero cultures were observed for CPE daily, and culture supernatants were collected at indicated times for WNV viral RNA extraction and TaqMan RT-PCR [17].

## Authors' contributions

SD carried out the WNV infection and protection assays including CPE observation; CC extracted viral RNA and performed TaqMan RT-PCR; QS, YH, and YL helped in preparing of NK cells and cytotoxicity assays, AG helped in WNV infection and detection; HW helped in the FACS analysis; MR participated in the study design and coordination; AD participated in the study design and coordination, and helped to revise the manuscript; MJ conceived, designed, participated, and coordinated the study, and wrote the manuscript. All authors read and approved the final manuscript.
